# Humanising processes after harm part 2: compounded harm experienced by patients and their families after safety incidents

**DOI:** 10.3389/frhs.2024.1473296

**Published:** 2024-12-17

**Authors:** Lauren Ramsey, Joanne Hughes, Debra Hazeldine, Sarah Seddon, Mary Gould, Jo Wailling, Jenni Murray, Siobhan McHugh, Ruth Simms-Ellis, Daisy Halligan, Katherine Ludwin, Jane K. O’Hara

**Affiliations:** ^1^ Yorkshire and Humber Patient Safety Research Collaboration, Bradford Institute for Health Research, Bradford, United Kingdom; ^2^ Patient and Family Advisory Group, University of Leeds, Leeds, United Kingdom; ^3^ Faculty of Health, Victoria University of Wellington, Wellington, New Zealand; ^4^ School of Humanities and Social Sciences, Leeds Beckett University, Leeds, United Kingdom; ^5^ School of Psychology, University of Leeds, Leeds, United Kingdom; ^6^ Research and Innovation, Midlands Partnership NHS Foundation Trust, Stafford, United Kingdom; ^7^ School of Healthcare, University of Leeds, Leeds, United Kingdom

**Keywords:** patient safety, patient involvement, compounded harm, healthcare harm, safety investigations, healthcare litigation, qualitative research

## Abstract

**Background:**

Healthcare organisations risk harming patients and their families twofold. First, through the physical, emotional and/or financial harm caused by safety incidents themselves, and second, through the organisational response to incidents. The former is well-researched and targeted by interventions. However, the latter, termed ‘compounded harm’ is rarely acknowledged.

**Aims:**

We aimed to explore the ways compounded harm is experienced by patients and their families as a result of organisational responses to safety incidents and propose how this may be reduced in practice.

**Methods:**

We used framework analysis to qualitatively explore data derived from interviews with 42 people with lived or professional experience of safety incident responses. This comprised 18 patients/relatives, 16 investigators, seven healthcare staff and one legal staff. People with lived and professional experience also helped to shape the design, conduct and findings of this study.

**Findings:**

We identified six ways that patients and their families experienced compounded harm because of incident responses. These were feeling: (1) powerless, (2) inconsequential, (3) manipulated, (4) abandoned, (5) de-humanised and (6) disoriented.

**Discussion:**

It is imperative to reduce compounded harm experienced by patients and families. We propose three recommendations for policy and practice: (1) the healthcare system to recognise and address epistemic injustice and equitably support people to be equal partners throughout investigations and subsequent learning to reduce the likelihood of patients and families feeling powerless and inconsequential; (2) honest and transparent regulatory and organisational cultures to be fostered and enacted to reduce the likelihood of patients and families feeling manipulated; and (3) the healthcare system to reorient towards providing restorative responses to harm which are human centred, relational and underpinned by dignity, safety and voluntariness to reduce the likelihood of patients and families feeling abandoned, de-humanised and disoriented.

## 1 Introduction

Patient harm is a persistent, and seemingly intractable, international issue that has been widely researched [e.g. (1)] and is the target of widespread policy directives (e.g. Patient Safety Strategy, NHS England), biomedical interventions [e.g. (2)] and improvement initiatives [e.g. (3)]. Substantial attention and resources are afforded to reducing avoidable patient harm. In England, this includes the Health Services Safety Investigations Body (HSSIB), NHS Resolution, the Parliamentary and Health Service Ombudsman (PHSO) and the Care Quality Commission (CQC). Indeed, Oikonomou et al. (4) revealed over 126 organisations that exert regulatory influence on NHS provider organisations to improve the quality and safety of care. Nonetheless, efforts to systematically reduce avoidable harm have been impeded by an increasingly complex and adaptive landscape facing various challenges, such as a growing and ageing population, increasing rates of comorbidities and mental illness, the rising use of digital technologies and a push to virtual home care. In cases of avoidable harm, investigations to explore what happened, how it happened and what can be learned to reduce the risk of it happening again are cornerstones of international patient safety policy. For example, the Patient Safety Incident Response Framework published by NHS England (5) is underpinned by a need for organisations to learn and reduce future avoidable harm.

However, there has been a growing recognition of the failure of organisational responses to acknowledge the wide-ranging human impacts on those affected, which can sometimes feel worse than the original harm itself (6–8). In addition to the initial harm resulting from patient safety incidents, ‘compounded harm’ can extend the harmful experience for everyone involved (9). Compounded harm refers to the harm that can be created after a safety incident, due to the processes that follow by ‘neglecting to appreciate and respond to human impacts’ and has been argued to be especially the case ‘when people feel unheard or invalidated’ (7, 10). Bismark and Paterson (11) proposed that organisations should respond in accordance with four simple sayings: honesty is the best policy, say sorry if you hurt someone, we can all learn from our mistakes, and treat other people the way you would like to be treated. While these represent modest moral bases to inform organisational responses to incidents, over five decades worth of well-documented care failings demonstrate that they do not always translate into practice [e.g. (12–16)]. The PHSO also recently suggested that compounded harm is ‘often neglected in the process of understanding the impact of avoidable serious harm’ (6).

Supportively, Wiig et al. (17) suggested that ‘respect, dignity, listening, and good relationships are all crucial for a wholistic and sustainable approach to care’ (18, 19). It has also been found that most patients and families value being involved in investigations of harm; however, it is important that investigations are flexible and sensitive to both clinical and emotional aspects of care (20). This literature review highlighted important factors including early active listening with empathy for trauma, sincere and timely apology, fostering trust and transparency, making realistic timelines clear and establishing effective non-adversarial communication. McQueen et al. (21) also suggested that meaningful involvement in investigations can help with reconciliation following a traumatic event and help restore faith in the healthcare system.

Warranting further attention is the extent to which patients and families should and could be involved. Smits et al. (22) developed a potentially useful model to consider this via categorising patient involvement from listener (i.e. given information), through to advisor (i.e. gives unsolicited advice) and partner (i.e. works as an equal). Vincent et al. (23) suggested that there should be an assumption that patients and families will be active partners. However, NHS England is built on a foundation of paternalism (24), which began to shift in the 1970s and 1980s as patient perspectives, skills and expertise began to be acknowledged as valuable and untapped resources (25–27). This period saw the early UK patient campaigns for increased autonomy in mental health, disabilities and maternity care (28–30), and the radical notion of coproduction being developed (31, 32). Hereafter, healthcare policy has made increasing promises to involve patients and their families as partners (e.g. 33–35), with the introduction of more recent initiatives such as Patient Safety Partners being rolled out in NHS England. The movement has also been fuelled by historical cases of overlooked warning signals raised by patients [e.g. (36)]. Martin (37) outlined two key rationales for involving patients and families – firstly, because it is a moral obligation of the health service and, secondly, because it provides otherwise omitted and clinically useful information [e.g. (38)].

To advance the current evidence base, we aimed to explore the types of compounded harm experienced by patients and their families as a result of organisational responses to patient safety incidents and propose how compounded harm may be reduced in practice.

## 2 Methods

A favourable ethical opinion for this interview study was received in July 2020 (REC Ref. 20/EE/0133). Interviews took place between September 2020 and April 2021. Participants were recruited using a targeted sampling approach to gain interest from those who had experienced a patient safety incident and subsequent investigation as a patient, relative, healthcare professional, investigator or legal staff within the United Kingdom. Interested people contacted the research team via email, were provided with an information sheet (easy-read when preferred) and were assessed for eligibility via telephone. Criteria stipulated that participants must be >16 years old, have experienced a ‘serious incident’ and subsequent investigation within a healthcare setting as defined by the Serious Incident Framework (39), have experienced the serious incident >1 year after consenting to take part, have no related ongoing police or legal involvement relating to the incident and have the capacity to consent. Eligibility assessment followed a detailed semi-structured guide. Participants were signposted to personalised sources of support where necessary.

A total of 117 people registered interest, 98 people were assessed for eligibility, and 66 were eligible, of which 42 consented to participate. Forty-two people with lived or professional experience of incident responses took part in individual and virtual semi-structured interviews with one of four researchers (LR, KL, RS-E, SMcH). Interviews were supported by a topic guide which enabled avenues of conversation to remain focused on the research questions, while also allowing flexibility to capture wider topics of interest, including exploring topics most important to participants themselves. Topic guides were tailored for each stakeholder group; however, all questions centred on experiences of incident response processes, their thoughts and feelings about, and experiences of, involvement and their experiences of interlinked processes including decisions to litigate. Interview duration ranged from 25 min to 2 h 32 min (average, 1 h 27 min). Further details of the recruitment strategy and interview methods are described elsewhere (40), as well as details of the primary analysis and findings which explored and compared the experiences of stakeholders including patients, their families, healthcare staff, investigators and legal staff. This paper focuses specifically on a secondary analysis, in which interview data were reconsidered to provide a considerably distinct perspective.

### 2.1 Analysis

The research team comprised four harmed patients/relatives whose experience related to physical (*n* = 3) and mental health (*n* = 1) care and eight health services researchers with disciplinary backgrounds in psychology (*n* = 4), sociology (*n* = 1), nursing (*n* = 1), applied sciences (*n* = 1) and medicine (*n* = 1). The 42 interviewees included six patients directly affected by the incident and 12 relatives. The 18 patients/relative experiences related predominantly to acute (*n* = 13) and mental health care (*n* = 5), although some spanned multiple settings and others also related to separate investigations or inquiries (*n* = 2) and were completed by an independent investigatory body (*n* = 1) rather than by a Trust locally. Incidents included delayed/misdiagnosis (*n* = 6), surgical error (*n* = 4), maternity harm (*n* = 2), suicide (*n* = 3), drug error (*n* = 1) and unexplained death (*n* = 2). Of the 18 incidents, 14 resulted in severe harm or death. The seven healthcare staff interviewed worked within acute (*n* = 5) or mental health settings (*n* = 2), and the 16 investigators interviewed worked in acute (*n* = 3), mental health (*n* = 7) and national settings (*n* = 5). One worked across settings as a bank investigator. One member of legal staff was also interviewed.

A secondary analysis was conducted as a broad thematic approach to qualitatively analysing data. This can arguably dilute the specific meaning of experiences for individuals and within the context of their own life. Sometimes this is overcome by conducting rich analyses such as case studies (41). However, typologies can bridge the gap between within-case and cross-case approaches. Mandara (42) defines a typology as a ‘system of categories used to organise objects according to their similarities and dissimilarities’. Therefore, framework analysis was used to identify the types of compounded harm experienced by patients and families following an iterative process (43, 44).

First, data were transcribed via Zoom or Teams software initially where possible and, after checking, were transcribed verbatim. Second, the authors involved based on their lived experience of safety incidents (DH, MG, SS, JH) underwent a basics in qualitative research training session focussing particularly on framework analysis (delivered by LR with support from Dr Giorgia Previdoli). Third, two researchers (LR, DH) extracted all interview data relating to compounded harm to Excel, whereby compounded harm referred to not the original harm of the incident, but harm created after due to the processes that follow (9). Due to the nature of the research questions, this related to compounded harm experienced by patients and their families only, but from all interviewees’ perspectives. A total of 672 excerpts were extracted for coding. Fourth, extracted data were thoroughly read multiple times to gain a holistic view, noting descriptive initial impressions as well as convergence and divergence. Discussion between authors, focusing on the significant and common features across the data, led to a provisional coding framework being developed. Iterative discussion between authors led to ongoing refinement of the framework until a consensus was reached. One researcher (LR) then systematically coded the data according to the agreed framework, with significant data helping to define and further refine each type. Where it was deemed appropriate, data were coded multiple times. Ten per cent of anonymised data were independently coded a second time to ensure consistent interpretation and application of the framework. Finally, a matrix was developed to summarise the titles, definitions and number of cases. All data sources were included in the analysis; however, the representation of data sources was not necessarily equal, and all sources were not necessarily represented but included depending on data relevance, quality and significance.

## 3 Findings

**Figure 1:**
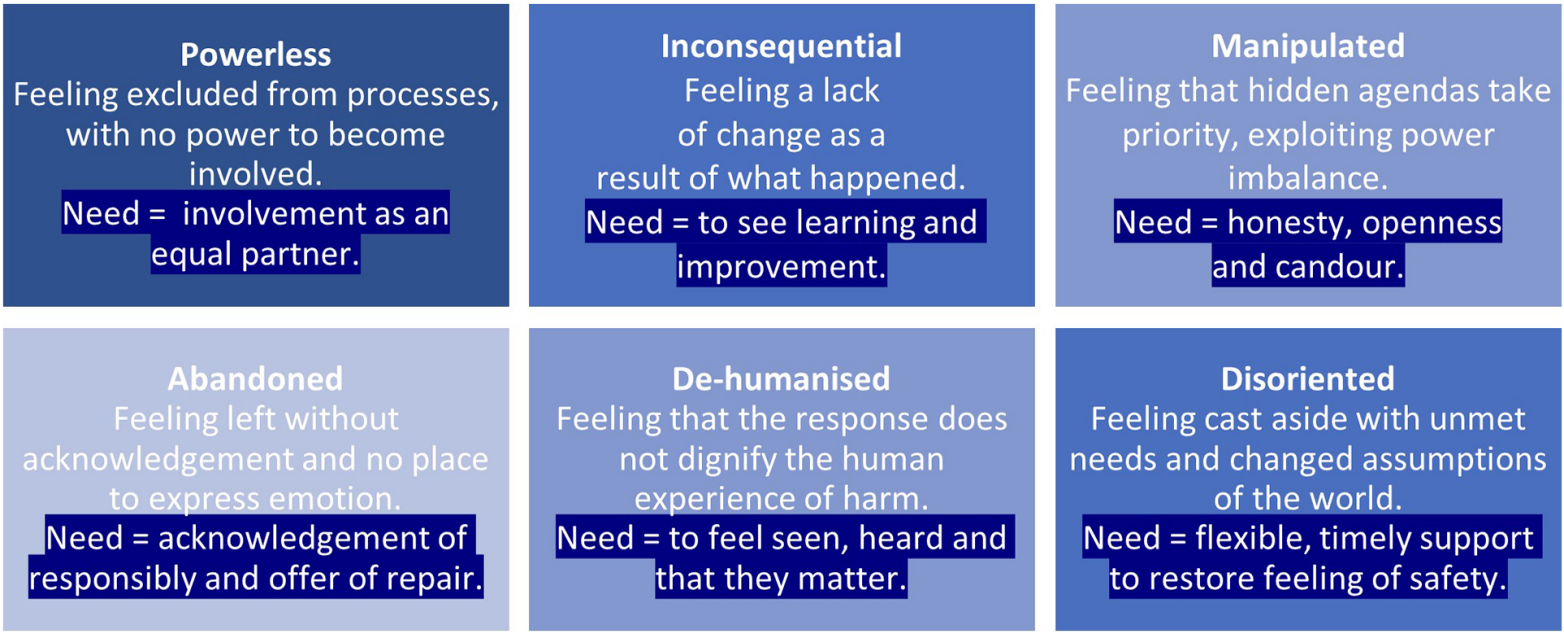
Six types of compounded harm experienced by patients and families and the underlying need.

Authors extracted a total of 672 interview excerpts relating to compounded harm experienced by patients or their families as defined by Wailling et al. (9). These data represented 39 of the 42 interviewees, as three staff interviews did not refer to this concept. Table 1 provides a summary of how data were coded for the purpose of transparency, rather than to indicate statistical significance. Based on these data, a typology was developed outlining six key types of compounded harm that patients and their families experienced following safety incidents. The types centred on feeling (1) powerless, (2) inconsequential, (3) manipulated, (4) abandoned, (5) de-humanised and (6) disoriented. The 672 excerpts were coded a total of 721 times, as 33 excerpts were coded multiple times (see Figure 1 for a summary of the types of compounded harm).

**Table 1:** Data coded according to the typology of compounded harm experienced by patients and families.

	Powerless	Inconsequential	Manipulated	Abandoned	De-humanised	Disoriented	Other	Total
Total no. of codes	147 (20.4%)	38 (5.3%)	179 (24.8%)	74 (10.3%)	98 (13.6%)	178 (24.7)	7 (1.0%)	721
No. of codes from patient/relative interviews	86 (15.8%)	35 (6.4%)	159 (29.3%	42 (7.7%)	66 (12.2%)	151 (27.8%)	4 (0.7%)	543
No. of codes from staff interviews	9 (22.0%)	0 (0.0%)	9 (22.0%)	5 (12.2%)	14 (34.1)	3 (7.3%)	1 (2.4%)	41
No. of codes from investigator interviews	47 (36.2%)	3 (2.3%)	11 (8.5%)	27 (20.8%)	18 (13.8%)	22 (16.8%)	2 (1.5%)	130
No. of codes from legal interview	5 (71.4%)	0 (0.0%)	0 (0.0%)	0 (0.0%)	0 (0.0%)	2 (28.6%)	0 (0.0%)	7

**Table 2:** Interview data relating to feeling powerless.

‘They’re left there knowing that there's an investigation but they’ve no control…they’ve no control over the findings, they’ve no control over what happens afterwards, you know, the sense of control - it seems, they don't have any’. (Investigator)
‘Why didn't they engage with the family more, who know this person, who have lived with this person for 30-odd years?’ (Relative)
‘She couldn't understand why, you know, six or seven months after her partner's death, someone was suddenly ringing her up… I found it difficult because her concerns weren't part of the actual purpose of the investigation. When I tried to explain, no, you can't include that in the investigation… the lady actually specifically said to me, you know, “No one has involved me in this, you’re ringing me up with this process I knew nothing about, no-one's involved me, no-one's listened to me, no-one's talked to me.” So, I found the whole thing really difficult’. (Investigator)

**Table 3:** Interview data relating to feeling inconsequential.

‘Part of me is like, did the outcome mean nothing? It was brushed off’. (Patient)
‘I personally struggled with the report… it didn't dig deep enough… there was so much information under the system, questions that didn't get asked… “she was offered medication, she was offered this, she was offered that, oh, we've got nothing to learn then, really.” We've always got things to learn…’ (Staff)

**Table 4:** Interview data relating to feeling manipulated.

‘It's all so cloak and daggers, isn't it. Professionals are so scared that if they admit anything they’re going to get done, and so everyone's so hush-hush about it and it's wrong, it should be so much more open’. (Patient)
‘I have found that the, the untrustworthy nature of my experience is not unique… I have found hundreds of people who had the same kind of story to share of fraudulent amended medical records, flat-out lies, evidence disappearing, you know, twisted language to try and create an impression of one thing when it's really another. It's a cesspit… the exact opposite of what I expected to find’. (Relative)
‘We were denied [the truth] by a secretive Trust that wanted to cover their own backs and that should not be. It's happening now, we know it is because we meet families, and that's got to stop’. (Relative)
‘All they were doing, they were covering their arses and preventing legal issues. Or, you know, minimising the cost to the Trust, and that unfortunately creates a world that is not right or fair’. (Patient)
‘They denied ever sending me this letter, but they did. When I went back for my review, all my notes were missing. Somebody had shredded some of the files, deliberately lost files, misplaced files. So, I had four volumes, apparently, of my records, and I now only have two, you know, a massive cover-up. There were two conflicting reports, both with the same report number. So, for me, it turned into years of this fight, (1) to get the truth for myself and then, (2) to get the truth for other people’. (Patient)
‘It's not easy when you're amongst these professionals who think they know it all. History has shown that they don't know, they don't know it all’. (Relative)
‘There was always like a bit of doubt with that case because when you meet with clinicians, you can only rely on what they're telling you, and, you know, sometimes I kind of got this feeling that, are you all closing rank? you know, is there something, you know, that, we didn't have enough evidence to know what went on, on that shift when she died… there was always this little seed of doubt in my mind, you know, but you're limited, if you haven't got the evidence, you haven't got the evidence… that doesn't feel great’. (Investigator)
‘The trust said, ‘Oh no, the records show that…”. And it's that kind of, and I'm going to say it quite bluntly, that kind of stupidity… I've then got to say to the family that they don't know the difference between a man and a woman… to suggest that their memory recall is less accurate than the staffs’ and that the clinical records are always 100% correct and accurate. It is utterly ridiculous’. (Investigator)
‘I've worked with probably half a dozen or so families in the last 16 years who stick in my mind, who have been given a really bad time by the NHS, labelled as vexatious when they have not been. Not given honest answers to their questions… they've been given the run-around… the way an NHS Trust has written its report… was either not at all clear or had been worded in a way that wasn't an exact lie but was also disingenuous… you can't have a half-truth if you're working in an open and honest, transparent way with a family’. (Investigator)

**Table 5:** Interview data relating to feeling abandoned.

‘You almost think, well, have they forgotten about us? That's how it kind of feels’. (Relative)
‘I wrote a really carefully worded complaint letter. I put a lot of thought into it, even though, you know, I did feel angry and upset, I tried to make sure that the letter wasn't aggressive or pointing the finger… but when I got the response, I've often said that was the worst day of my life’. (Patient)
‘As a Trust we don't have designated bereavement support for an unexpected death… as a team we’ve got families who, you know, you just feel that they’re left floundering… you just sometimes feel it's only a Level 1, they’re nothing to do with us, but actually they’ve been involved in an incident’. (Investigator)
‘It seems that the focus is on getting that duty of candor letter out within this timeframe and then obviously, you know, the ward have moved on, the investigation has been declared and it's now in the hands of an investigator’. (Investigator)
‘If we don't engage with the families correctly, we lose the opportunity to retain trust and faith in the NHS and we lose the opportunity to help families in a healing journey following avoidable healthcare harm. Just simple as that… We're not counsellors, we’re not therapists, we're not there for that. Definitely not our role, but I think it can help and assist if a situation is managed correctly, or you can do great harm’. (Investigator)

**Table 6:** Interview data relating to feeling de-humanised.

‘There was a bank of journalists outside the court wanting to photograph me and a friend who was a victim and another lady that was there, and they thought it was great to just photograph us all crying, you know, and really upset’. (Patient)
‘It was like a conveyor belt at times… I could have eight or nine reviews on the go… you’re ready to explode with all the balancing it all. I'd say it was the hardest role I’ve ever done actually’. (Investigator)
‘What really shocked me was when I went back to the family home to deliver the report… I think it was eleven months later, this baby, she was a big baby… but it wasn't doing anything… it was just laid there, looking up, vacant… having to be tube fed through the nose, and for the first time I thought, I’ve never even considered the babies that are affected. This is the life this family's got, and it shocked me, it upset me, because I came away thinking, ‘Why have I never?”… because I’ve never seen it… the implications and the effect it had on that family… That's really helped me looking at cases now to remember there's a family, there's a baby, and why we’re doing this and why it's so important’. (Investigator)

**Table 7:** Interview data relating to feeling disoriented.

‘The only way I could get what had happened to me in writing, really, was to go and see a solicitor and get an independent review of my medical notes. So that's what I did… it was like a breath of fresh air, actually being told the details, it was such a massive relief, because I felt like I was going mad. You know when you know inherently there's something wrong because you can hear enough information, but you can't join all the dots, nobody's joining the dots for you, you've got to try and eke out like little bits of information, and then to actually read that I didn't need [the procedure]’. (Patient)
‘Often you don't need, you don't want to make a complaint, you just want to be acknowledged that it happened’. (Patient)
‘People take legal action as a last resort because of the defensiveness of the Trust’. (Relative)
‘We’d got a final report, and the father wasn't happy… I felt I had no option but to ask him to resubmit it as a complaint… because that was handled in a different way… they would have had a point of contact… it would be looked at by our head of governance… a better process than we had for the actual investigations… the matron would have a family meeting where we’d go through and literally all the questions we answered, and if the family weren't happy about anything then it would be looked at, but that's what we weren't doing with our investigations’. (Investigator)
‘We come up with recommendations and we come up with an action plan. And that's shared with the family. But then it's like they fall off a cliff… we are improving our services, and we are learning lessons… I think we kind of need to demonstrate that to the, the people that have helped, you know, influence that process… But I don't know whose role that is’. (Investigator)

Examples of interview data classified according to the typology are provided both within the detailed explanations of each type of compounded harm and within a separate table (see Tables 2–7).

### 3.1 Powerless

This type of compounded harm refers to patients and families feeling excluded from investigation processes, with no power to become involved (see Table 2 for additional data). Patients and families described feeling without strength, ability or power to act, influence or prevent things from happening throughout investigatory processes.

The whole process went on as though we didn't exist… If we hadn't have been persistent, to this day it would have all been brushed under the carpet. Totally, we were not in the picture. (Relative)

Initially, most patients and families were overwhelmed while managing the physical, emotional and/or financial aftermath of the incident. This meant that their abilities to proactively ‘reach in’ to the investigatory system were compromised. Having never been through an investigation of a patient safety incident before, patients and families also felt unequipped and described expectations of individuals within the system to instead, proactively ‘reach out’ and support them in due course. On that basis, most proceeded in good faith and put their trust in the staff they initially encountered. Some described being given false promises of involvement that never materialised and were later left to make sense of why they were not being supported, with some questioning if staff were busy making progress with the investigation without them, or if they were purposefully excluding them.

Don't just send me a report in the post and expect me not to have questions or not to want to discuss it. At some point, surely the patient should be brought in to have these things discussed… Talk to me. Include me. Don't sit in a room and talk about my situation behind my back and then send me a report. Let me be there. (Patient)

Over time, it became clear to families that they themselves had to ‘work’ in the absence of guidance and clarity to navigate the complex system designed without their needs in mind. Some underwent what felt like a ‘self-taught crash course’ in understanding investigation processes. Ambiguity surrounded elements including the indistinctness of the investigation itself and other interlinked processes (e.g. complaint, litigation, and inquest), how terms of reference had been determined and why certain elements were excluded, the roles and responsibilities of various personnel, and trying to understand what had already happened in the investigation process without their knowledge. For some, a key point in the investigation was receiving an investigation report. Reports often reinforced divergence in expectations and were described as using inaccessible language. For most, the report presented a narrative that was incongruent with the patients’ and families’ experiences and expectations. For example, for some, it provided ‘new’ information and, for others, it was the point at which they realised that promises had been broken and their questions had not been acknowledged or answered. As a result, this was also a difficult and uncomfortable stage in the process for investigators.

In terms of involving the family, it would be at the end of the report process. So, we’d have done the investigation. They might not have even known an investigation was going on, or the ins and outs of it. But once the report was ready, we would then offer meetings… And I remember sitting in meetings offering this final report, and the family being very upset. We hadn't achieved anything in terms of trying to answer family's questions, we hadn't even asked them what the questions were. (Investigator)

Frustration was also felt as the report was perceived to be accepted as an objective truth, with no right of reply. It was often considered too late to be meaningfully involved and influence the report in the ways many would have liked to with hindsight rendering a sense of powerlessness. Ultimately, people needed to be offered to be involved as an equal partner, and for that offer to materialise for those who wanted it to.

### 3.2 Inconsequential

This type of compounded harm refers to feeling a lack of change as a result of what happened (see Table 3 for additional data). For some, needing reassurance that there was organisational learning and that the same would not happen to others in the future was a key motivation. However, many described neglected opportunities to learn.

I just feel that yes, I went through this and anyone else can go through it again afterwards. No one's learnt anything from it. (Patient)

This centred on investigation blind spots because of misaligned and narrowly focused inquiry, restricted opportunities to look back at care history and failure to take account of cross-setting interactions with services. Others raised concerns of history repeating itself, as the issues seen in care then became intertwined within the investigation, such as poor communication, delays and evasive reporting. Some perceived that this was due to procedural constraints, whereas others surmised that the organisations chose not to see and confront issues head-on but rather circumvent the real issues that needed attention. Others raised concerns of arbitrary recommendations that did not indicate that organisational learning would take place. One patient described how they had to revisit the same care setting again for a similar procedure and witnessed first-hand that a recommendation had not been actioned. Similar frustrations were expressed by investigators and healthcare staff.

You can't go back to the family and say, right, I’ve done this, this is what's going to happen because you’ve got no idea whether any of it is actually going to happen. I know we’d like to think that once we’ve done it everything that you suggest is going to happen, but you know full well that, you know, some things can happen, and other things can't, and some things will and others won't. (Investigator)

Ultimately, people needed to see learning and improvement.

### 3.3 Manipulated

This type of compounded harm refers to patients and families feeling that hidden organisational agendas take priority, exploiting power imbalance (see Table 4 for additional data). All stakeholders felt that investigatory systems were built on an assumption of honesty and good intention; however, most perceived a degree of manipulation as the process unfolded.

She was very guarded, and I just felt that all the staff that were interviewed were protecting their position. And at that point, it was very obvious that they were closing ranks. (Relative)

Patients and families, in general, felt that investigation processes were not set up to meet their basic needs but, instead, aligned with organisational needs. Factors that raised concerns included lacking transparency, contrived communication, limited investigation scope and the instigating of adversarial relationships.

But it's like the terms of reference of an inquiry, they're sometimes set out not to get to the truth. (Relative)

Despite understanding why patients and families may feel manipulated in such circumstances, similar concerns were also raised by investigators and healthcare staff.

It's in the bones of the NHS… there's so much covering of backs, it's so in-ground in our system, and I can really understand that, especially because I've just been investigated [laughs]. You know, you can be struck off, so I can really understand it, but I think it can get in the way of meaningful conversation and dialogue with people because of this fear of litigation. (Staff)

Some felt that the timeline of investigations strategically dissuaded people from seeking answers and others felt accused of causing the outcome themselves. Reports were described as disheartening, disrespectful, dishonest and defensive. Many felt that the report withheld, concealed or covered-up information, and others felt that the information they had desperately waited for only became available due to organisational deadlines. Frustration was also felt when staff within the organisation privately raised concerns but were not prepared to go on record for fear of disruption and personal consequences. There was a perception that more weighting was given to protecting the organisation, than objectively understanding what happened from all perspectives.

As time's gone on their behaviour continues to show that they're not listening and not engaging… it feels that their priority is one of, yeah, defending themselves as opposed to learning and listening to families. (Relative)

Some were discouraged that the investigation happened internally which was perceived to deny real scrutiny. The few that were happy with the report tended to feel that it was not designed with them in mind, but for the organisation. Some described how only with hindsight was it clear how power imbalances had been exploited. Being forced to live with perceived naivety and unfair treatment weighed heavily for some. For some, these issues were offset by having a single point of contact, knowledge and support. Some called for an established advocacy role with relevant skills and knowledge. Ultimately, people needed honesty, openness and candour.

### 3.4 Abandoned

This type of compounded harm refers to patients and families feeling left without an acknowledgement of responsibility, often centred on absent or insincere apologies (see Table 5 for additional data). Following a patient safety incident, a shift in relationship dynamics with the healthcare system was described, sometimes sudden and obvious.

Nothing like “we’re really sorry that this is happening to you, and we’ll do our best to sort it”, it wasn't like that at all… it didn't feel like anyone was like holding my hand through it. (Patient)

For others, the diminution of the duty of care was more subtle. These breakdowns in relationships were manifested in a variety of ways, including failing to acknowledge the potentially profound and permanent impacts of the incident and the increasingly adversarial nature of communication. Often, people felt that their emotion, distress or anger was an unwelcome complication, despite what had happened.

We were grieving. No one actually realised that… No support at all. (Relative)

Patients, families and staff also raised the potential therapeutic value in coming together to set the basis for mutual understanding, apology and healing; however, most experienced a cease in communication with direct care providers due to organisational policy. From a patient and family perspective, compassionately attending to these needs was considered a basic step towards making amends when something had gone wrong. These experiences jarred with, and in some cases shattered assumptions, understandings and expectations of what caring organisations were supposed to do, particularly at a time when they relied upon them most.

You want some sort of apology. Not necessarily an apology of what they’ve done wrong. I mean yes that would be brilliant. But even just an acknowledgement that you’ve gone through an awful time… Getting their letters I just cried because it's so, so awful. They were so unfeeling, so apparently uncaring. (Patient)

Some suggested that this change was driven by a culturally engrained fear of litigation or blame for individual staff and teams, as well as wider reputational concerns. Others suggested that there was a lack of clarity surrounding whose role and responsibility it was to engage with the patient and family and a lack of support system for them to do it well.

There's always that sort of confusion around who should be involved, from the staff team, with the relatives. You know, so when you get that phone call of, say, someone's taken their life or someone's been killed, if you've been working with them you just want to go and see them… often then the managers come in and then they're the ones that have contact, because it just goes into the policy of, right, it's a serious incident, so A, B, C and D happens… It might say it on the policy, but that should only be a guideline. (Staff)

Ultimately, people needed an acknowledgement of responsibility and an offer of repair.

### 3.5 De-humanised

This type of compounded harm refers to patients and families feeling that their dignity was not supported or maintained, where dignity refers to a value-based and humanistic concept that demands respect for the integrity of human beings and their beliefs (see Table 6 for additional data).

There's no human element in there at all. It's just words on a piece of paper typed up by somebody and thrown in the post and that's the end of it really, you know, that's it. (Patient)

Examples included an unrequited desire to make sense of things most important to them, a lack of space to voice their needs, perceived careless inaccuracies in written and verbal communication such as copied and pasted information and typos, the insensitive delivery of unexpected information and being forced to live with unanswered questions.

I thought oh my god, you know, this is my life they’re talking about and the first I know that mistakes have been made is an apology through the post, not even a face-to-face, just a random letter through the post. Like I’m some kind of pack of meat on the supermarket shelf. I was gobsmacked. (Patient)

Patients and families also felt that staff and investigators often wanted to circumvent difficult conversations which made things worse, such as avoiding mentioning the name or death of a patient. These factors indicated organisational ambivalence about the most important thing to them, and that sight of the affected family had been lost, devaluing the experience they had been through. It appeared that while the family suffered sometimes life-changing consequences, the incident was insignificant to the organisation and did not demand the care and attention they felt it deserved.

He died and he'd been an impatient and his belongings had just been like literally, bundled together in a black bin liner. That looks like a little thing to a busy staff member, and obviously they didn't think, but this is the last time she's going to be given his belongings back, and they were sort of chucked in a bin liner. (Staff)

One family described how they felt they were not treated as a human being, but a ‘cog’ in a process. Overall, people felt that investigation processes were experienced as a challenge, during a time of sometimes extreme vulnerability, which was felt as de-humanising. However, from an investigator perspective, many did not have adequate protected time within their job plan, or appropriate skills, to engage with families in the ways that they needed.

There's nothing worse than somebody ringing you and started talking about a death and you’ve absolutely no idea what they’re talking about because you’ve just got so many. (Investigator)

Ultimately, people needed to feel seen and heard and that they and their experiences mattered.

### 3.6 Disoriented

This type of compounded harm refers to feeling cast aside with unmet needs, resulting in changed assumptions of the world (see Table 7 for additional data).

I am not the same person that came into this … I view the whole world very, very differently as a result of this experience, and I know that sounds very profound, almost an over-exaggerated thing to say, but I assure you it's not. I was somebody who would always have a default position of trusting somebody until they gave me cause not to… now I am exactly the opposite. (Relative)

Investigation reports marked a conclusion for organisations, yet often left patients and families in a state of disorientation that continued to torment them. As a result, some no longer trusted health services. For others, a lack of trust affected their worldview more widely, eroding their basic trust and sense of safety.

This is an unfinished journey for me… but I had to sort of step back and take a breath, and when I looked around me to all the years I'd spent… the rest of my life crumbled around me. (Relative)

Dissatisfied, many felt forced into additional procedures they hoped would be able to respond to their unmet needs. This included complaints, litigation, escalating via a local member of parliament, independent inquiries, seeking clinical advice and connecting with other patients and families affected by incidents. Often, decisions were not financially motivated, but people felt forced into finding new ways of meeting their needs.

I felt like I got pushed towards the legal approach because I didn't want money, like, you know, this wasn't about that. This was about getting a proper investigation. (Relative)

For some, this was an exhausting, emotional and lonely journey that had a ripple effect on wider aspects of their life, which were sometimes already in turmoil because of the incident, e.g. loss of career, lifelong disability, loss of identity, ongoing treatment, disruption of family dynamics, trauma, fear of revisiting services and mental health decline. Some also described how what happened became a taboo topic; how being drip-fed information then raised more questions for which they sought answers; or how they felt defeated by the process they felt forced to engage with.

I have this big, massive guilt complex, to think that all of this stress could have caused [my daughter] to have cancer, you know, it might seem illogical, but that's what gets me, is that it could have affected my kids. I had to say to [my son], look, I'm really sorry, I know it's our holiday, but I need to know what's going on in the court. I can't settle. He was caught up in it and obviously worried, they’ve all been worried about me. It's had a massive impact on my husband, everything. It's been really, really tough. People do say to me, you need to give it up now [laughs]. I suppose with me there's an element of, it’s still anger, I guess. I try not to let it rule my life, but it's been quite all-encompassing. It's about fighting for, you know, rights. But I have to pace myself because I don't want to stress myself out totally… I've been asked by the NHS, can I refer this patient to you? This is in my own time… nobody pays me to do this. I also have my own time and my own things that I want to do, but they do refer people to me, and I'm thinking, it's probably not right, but who else do they go to? (Patient)

Families perhaps felt particularly beholden to continue in what felt like a quagmire of hope that something meaningful would come of their efforts, if harm resulted in the death of a close loved one such as a parent, spouse or child. This was an ongoing internal conflict for some who felt a sense of obligation to continue fighting for their loved one who had been harmed, but also owing to themselves to step back. This internal conflict sometimes occurred over a protracted period as they became stuck in a cycle of investigation, feeling forced to keep what happened in the forefront of their mind and constantly reliving what happened. Some spoke about the emotional impact of becoming a support for other harmed patients and families yet feeling compelled to continue to do so in the absence of formal support. Ultimately, people needed flexible, timely support to feel safe in the world.

Of the 615 excerpts extracted, 7 could not be classified according to the typology described. Four of these were from patients/families, of which three were from the same participant. All four excerpts referred to the existence of an investigation making them feel more anxious and worried about what had happened. For example, ‘I've gone away, and thought, actually, maybe I have not taken it as seriously as I thought I should have done. And then that's a whole different thing. You think, actually that was really serious and then it kind of plays on your mind’ (Patient). One excerpt was from staff, and one of the two excerpts from investigators spoke to the same issue for example, ‘They were very perplexed by being phoned up. They couldn't understand why someone was contacting them about it… it seemed to be raking it up and going through things that they had spent a lot of time dealing with and coming to terms with… the perception was, well we’re saying this is a problem when actually the patient had come to the conclusion it's not a problem. You’re stirring a hornet's nest up’ (Investigator). The second investigator excerpt referred to patients and families requiring 24/7, 365 days a year support that they could not offer.

## 4 Discussion

In this paper, we present a newly developed typology of how compounded harm may be experienced by patients and families, as a consequence of investigatory processes that follow patient safety incidents. Our typology consists of six features of compounded harm, which leave patients and families feeling (1) powerless, (2) inconsequential, (3) manipulated, (4) abandoned, (5) de-humanised and (6) disoriented. This is an important advancement of the concept of compounded harm, a term which has gained a lot of traction recently in academic publications (9, 45) as well as guidance and policy documents [(6); NHS England, PSIRF]. It provides a delineation of the general concept and supports the development of interventions and approaches which specifically attempt to avoid or reduce different features of compounded harm. With this in mind, we present a set of recommendations for policy and practice (see Box 1) that combine our findings, with existing theory and empirical literature, before exploring three related key concepts – justice, restorative responses and accountability, in detail.

BOX 1 **Recommendations for policy and practice according to the types of compounded harm experienced by patients and their families.**

1. The healthcare system to **recognise and address epistemic injustice** (46) and equitably support people to be **equal partners** throughout investigations and subsequent learning (23), to reduce the likelihood of patients and families feeling **powerless** and **inconsequential**.
2. **Honest and transparent regulatory and organisational cultures** to be fostered and enacted (47), to reduce the likelihood of patients and families feeling **manipulated**.
3. The **healthcare system to reorient** towards providing **restorative responses** to harm (9) which are human-centred, relational and underpinned by dignity, safety and voluntariness to reduce the likelihood of patients and families feeling **abandoned**, **de-humanised** and **disoriented**.

### 4.1 Justice after harm

Research has suggested that there are multiple justice lenses that should be considered in the aftermath of healthcare harm (48). One interesting lens through which to look at our findings is that of epistemic injustice (46), which is of significance for patients and families. Epistemic injustice is a way of understanding how people can be ‘wronged’ in the context of their capacity as a ‘knower’ (49). Asymmetries in power dynamics between patients, families and the health service were illuminated in our study, which materialised in both obvious and more subtle ways. For example, our findings highlight the sometimes crippling and limited space to express emotion after experiencing healthcare harm, leaving people feeling abandoned. This could perhaps be conceptualised as a termination of the duty of care and deemed antithetical to healing. This was despite well-intentioned investigators who felt unequipped to support people and a confusion and diffusion of responsibility. Concerns were also raised about compounded harm potentially being experienced more profoundly for those who experience other social injustices, evidenced in other fields of research due to factors such as systemic racism [e.g. (50)], poverty [e.g. (51)], disability [e.g. (20)] and religion or belief (52). Fricker (46) proposed that patients and families are prone to suffer epistemic injustice; for example, when their testimonies do not suit the structure of an investigation, they may feel quietened or silenced – also termed epistemic exclusion (53). Fricker conceived of two forms of epistemic injustice: (i) testimonial injustice which ‘occurs when prejudice causes a hearer to give a deflated level of credibility to a speaker's word’ and (ii) hermeneutical injustice which occurs ‘when a gap in collective interpretive resources puts someone at an unfair disadvantage when it comes to make a sense of their social experiences’ (49; p.1).

Drawing on theories of epistemic injustice, Kok et al. (54) explored responses to healthcare harm in the Netherlands and identified several structures in the incident investigation process, which can promote or hinder epistemic contribution in the process of incident investigations. Our findings support this work, which illuminated multiple instances of testimonial injustice to eliminate emotion from their testimony to heighten credibility, reporting that ‘the emotions that interviewed actors may have, are frequently framed as problematic for a team's fact-finding quest’ (54). What was also evident was the lack of a ‘right of reply’ to the established narrative of what happened contained within the final report (54), which has also been identified within mesh and maternity inquiries (7, 16). Adams, Maben and Robert (55) reported related findings in the context of healthcare complaints, where patients were thought of as ‘inexpert, distressed or advantage seeking’. Parallels of epistemic justice can also perhaps be drawn from social injustice seen in entirely different contexts, such as the Hillsborough disaster, Grenfell and the Horizon Post Office scandal. However, like Kok and colleagues, understanding the extent of hermeneutical injustice in this context is more difficult, as it would require evidence that testimony was deflated because of a conceptual deficit. Further research on epistemic injustice and its various forms would benefit from an explicit focus and longitudinal understanding of experiences of investigations over time and from different social statuses, both of which were beyond the scope of this study. Kok et al. (54) concluded that repeated calls to ‘involve more’ should be replaced with encouraging policymakers to be mindful of and address the structures that can cause epistemic injustice.

One viable way to recognise and address epistemic injustice, proposed by Fricker, is by ‘cultivating habits of virtuous listening’ (46), which is perhaps needed much more widely across the healthcare system, and not just following healthcare harm. Hicks (56, 57) argues that ‘relationships have potential to make us feel our best and to make us feel our worst’, and by honouring the dignity of others, we give rise to resolving conflict and rebuilding relationships that make people feel their best. Hicks defines dignity as ‘the mutual recognition of the desire to be seen, heard, listened to, and treated fairly; to be recognized, understood, and to feel safe in the world’. Herring (58) further suggests that one of the essential markers of ‘care’ is that it ‘expresses respect for the dignity of the recipient’. In the context of responding to harm specifically, Janoff-Bulman's work (59, 60) looked at traumatic life events and suggested that because of trauma, there is a loss of illusion and unspoken fundamental assumptions about the self and the world are shattered. Evident here is that when patients and families experience healthcare harm, their assumption that the healthcare service is inherently safe is shattered. Janoff-Bulman (59, 60) uses another term for disoriented and argues that subsequent ‘disequilibrium’ can force people to rebuild from scratch their internal conceptual system about the world and their place in it. In addition, undignified treatment in the wake of trauma – especially by those deemed responsible – can cause huge problems for this necessary rebuilding of internal conceptualisations, continuously compounding harm.

### 4.2 Restorative responses to harm

Restorative responses offer an approach to meet repeated calls to re-humanise investigation processes (7–9, 61, 62). Wailling and colleagues’ (9) argument – that ‘a restorative response is likely to reduce the level of compounded harm experienced by all the people affected’ and that the ‘risk [of compounded harm] may be reduced when investigations provide the opportunity for healing alongside models that seek system learning, with the former having been consistently neglected’ – has subsequently been further explored and evidenced (63). Underpinning a restorative approach is the recognition that ‘we are relational creatures all the way down, from the first moment of conception to the last gasp of death’ (64). Specifically, a restorative response aims to create safe and supportive conditions. Wailling et al. (65) proposed three justice needs as the basis of this approach (1) substantive needs, the actual harm that needs to be remedied; (2) procedural needs, the process of interacting, communicating and making decisions about the harms; and (3) psychological needs, the way one is acknowledged, respected and treated throughout the process, ensuring those affected can honestly communicate their differences, concerns and potential similarities with each other in a safe way. A resotrative just and learning culture approach has been piloted in a mental healthcare context in Australia (66, 67) and England (68). The New Zealand Ministry of Health also applied a restorative approach to the context of surgical mesh harm (7). In arguing for the use of a restorative approach, Nickson and Neikirk (45) suggested that a fundamental principle is voluntariness, further supporting calls for divergent conceptions of justice to be acknowledged and considered (48).

Despite providing great promise, our findings suggest that a restorative approach is at odds with how investigatory systems currently operate in the United Kingdom, as all stakeholders in our analysis perceived a culture of manipulation following healthcare harm. As the most talked about type of compounded harm, it is essential to acknowledge and attend to the unspoken notion, not just in rare cases of intentional criminal acts but also in relation to well-intentioned investigatory processes of well-intentioned care. One possible way to alleviate feelings of being manipulated is investigatory working having independent oversight. For example, de Kam et al. (69) explored the perceived value of an external chair on incident investigation committees and concluded that they were both valuable and critical for impartial inquiry. However, New Zealand research concluded that when such ‘external or impartial’ institutional or professional responses are characterised by ownership of the harm, they can still be experienced as manipulative and compound the harm for all involved (63). Lewicki et al. (70) also noted the importance of apology, with key tenets identified as acknowledging responsibility and offering repair. Therefore, with or without independent oversight, substantive systemic cultural reorientation is likely required, supporting calls to foster honest and transparent regulatory and organisational cultures (47), as well as the appropriate underpinning personnel, training and resource. This cultural adaptation is not to be underestimated and indicates that there is still work to do following the publication of the Francis report over a decade ago (2013). Here, following devastating failings of care at Mid Staffordshire Foundation Trust, a total of 290 recommendations were made. These provided a clear focus on transparency and introduced a requirement for organisations to be held accountable for poor episodes of care. This included introducing the Duty of Candour as a standard for healthcare providers, meaning that organisations were legally obliged to be open and honest with patients and/or their families when something went wrong that had, or could, caused harm. Ultimately, further work is required to understand if and how a restorative approach should be embedded over time within the English healthcare system and if the restorative approach should run in parallel with the existing investigatory system arguably geared towards learning.

### 4.3 Accountability after harm

Another interesting lens to look at our findings is accountability. Whilst accountability is a commonly used term, the reality of what it means in practice is opaque. Over two decades ago, and in response to one of the key publications that prompted the patient safety movement in the United States and globally (Institute of Medicine, To Err Is Human), the Hastings Centre initiated a 2-year programme to explore the ethics of patient safety policy and improvement. One of the cornerstone elements of this work was to understand accountability. Sharpe (71) usefully described two important components of accountability – backward-looking and forward-looking. Backward-looking accountability is the act of taking responsibility for something that has already happened; accepting accountability for an outcome or experience (71). Forward-looking accountability refers to the roles, responsibilities and obligations of those who, in the case of patient safety incidents, might be tasked with repair. Sharpe describes ‘…whereas responsibility in the retrospective sense focuses on *outcomes*, prospective responsibility is oriented to the deliberative and practical *processes* involved in setting and meeting goals’ (71; p.14). It is clear from our analysis that whilst in the two decades since, healthcare has made some movement towards recognising the need for greater backward-looking accountability (e.g. Duty of Candour within the United Kingdom) – there is much to do to shape and sustain an infrastructure to understand and support obligations of health and social care in achieving forward-looking accountability.

## 5 Limitations

First, while the focus of this paper was compounded harm experienced by patients and their families specifically, we recognise that harm can be compounded for all stakeholders involved. Wailling et al. (9) refer to this in their definition of compounded harm, and it is explored in the primary interview analysis (40). Second, we have identified that epistemic injustice plays an important role, and a restorative approach underpinned by restorative justice shows promise. However, as indicated by Cribb, O’Hara and Waring (48), there needs to be more research to understand how people conceptualise justice in the health setting differently and to inform the development of systems. In addition, we recognise that, perhaps due to the self-selecting nature of the study, most patients and relatives who took part experienced severe harm or death and had a negative experience of their investigation. Therefore, further research exploring positive experiences of investigations and experiences relating to incidents such as near misses and mild to moderate harm is needed to inform policy and practice.

## 6 Conclusions

Our newly developed typology outlines six ways that compounded harm may leave patients and families feeling: (1) powerless, (2) inconsequential, (3) manipulated, (4) abandoned, (5) de-humanised and (6) disoriented. We argue that the health service would benefit from prioritising three recommendations: (1) the healthcare system to recognise and address epistemic injustice and equitably support people to be equal partners throughout investigations and subsequent learning to reduce the likelihood of patients and families feeling powerless and inconsequential; (2) honest and transparent regulatory and organisational cultures to be fostered and enacted to reduce the likelihood of patients and families feeling manipulated; and (3) the healthcare system to reorient towards providing restorative responses to harm which are human-centred, relational and underpinned by dignity, safety and voluntariness to reduce the likelihood of patients and families feeling abandoned, de-humanised and disoriented.

## Data Availability

The raw data supporting the conclusions of this article will be made available by the authors, without undue reservation.
